# Research into the interplay between EFL teachers’ professional identity and EFL students’ grit in Libya

**DOI:** 10.3389/fpsyg.2025.1597290

**Published:** 2025-11-24

**Authors:** Ayman Riheel Alnaas Taha, Behbood Mohammadzadeh, Muftah Hamed

**Affiliations:** 1 ELT Department, Faculty of Education, CIU, Nicosia, North Cyprus, Türkiye; 2 Department of English, Faculty of Languages, Omar Al-Mukhtar University, Shahat, Libya

**Keywords:** EFL teacher professional identity, language grit, university students and instructors, relationship, Libya

## Abstract

**Introduction:**

This study aims to examine the interplay between EFL Teacher’s Professional Identity and EFL Student’s Grit in Libya with a particular focus on how students’ language grit is affected by teachers’ professional identity.

**Methods:**

The study employed language grit and teacher’s professional identity scales which were utilized to gather data through an online questionnaire administrated to 282 respondents (240 students and 42 teachers) from two government universities located in Aljabel Alakhder, eastern Libya. The link between students’ language grit and the professional identity of English language teachers was examined via Pearson’s correlation coefficient.

**Results:**

The results revealed no significant positive association between students’ language grit and English language teachers’ professional identity. Such results indicated that English language teachers’ professional identity was not one of the original sources of students’ language grit.

**Conclusion:**

The findings suggest that future educational initiatives could concentrate on enhancing teacher training and professional development and curriculum design to more effectively promote students’ perseverance in language learning. In addition, incorporating humanistic and contextually aware teaching methods may cultivate more enduring motivation and engagement among language learners in Libya.

## 1 Introduction

Over the past decades, research concerning second language acquisition (SLA) has increasingly drawn upon the principles of positive psychology to illuminate the emotional and motivational dimensions of language learning (Dewaele et al., 2019; MacIntyre and Mercer, 2014). Within this framework, grit—originally conceptualized by Duckworth et al. (2007) as passion and perseverance for long-term goals—has been adapted to the field of language learning as language grit. Language grit is defined as passion and perseverance for second language learning (Teimouri et al., 2022). Language grit thus represents both persistence in the face of challenges and consistent interest in language learning goals, aligning closely with positive psychology’s emphasis on learners’ resilience and well-being. MacIntyre and Khajavy (2021) considered second or foreign language student grit as an individual’s perseverance and determination to overcome the difficulties and challenges inherent in language learning with the definitive goal of achieving mastery of the target language.

As achieving proficiency in a second or foreign language largely depends on learners’ sustained effort (Teimouri et al., 2022), several studies have indicated that grit plays a positive and crucial role in predicting learners’ motivation, engagement, and overall achievement in English language learning (Teimouri et al., 2022; Teimouri et al., 2024; Zhang et al., 2022; Zhao and Wang, 2023a). However, few studies have reported no significant association between learners’ grit and their language outcomes. For instance, Alamer (2021a) found no link between grit and English vocabulary knowledge, while Khajavy et al. (2021) similarly observed no correlation between university students’ L2 achievement and grit. Such inconsistent findings may be attributed to variations in educational backgrounds, participant characteristics, and research contexts, as well as the potential influence of other underlying constructs.

Considering the antecedents and outcomes of language grit, little research has explored its connection with affective and interpersonal teacher-related variables. For example, Bai and Zheng (2024) examined the role of emotional support and teacher confirmation, while others have focused on teacher support (Hejazi and Sadoughi, 2022; Tang and Zhu, 2024), teacher support and respect (Shen and Guo, 2022), teacher stroke and teacher–student rapport (Yuan, 2022), and teachers’ enthusiasm, support, appreciation (Derakhshan et al., 2025). Overall, these studies highlight that learners who perceive their teachers as supportive, respectful, enthusiastic, appreciative, and emotionally encouraging are more likely to sustain higher levels of grit in language learning. Moreover, positive teacher–student rapport and the provision of affirming teacher strokes—such as verbal praise, recognition, or empathetic gestures—foster a sense of belonging and emotional security in the classroom, which in turn enhance learners’ persistence in the face of linguistic challenges.

While previous research has primarily focused on learners’ internal attributes, little attention has been given to the contextual and interpersonal factors that may influence or sustain students’ language grit. Among these, teachers’ professional identity stands out as a potentially significant yet underexplored factor. Teachers’ professional identity—broadly defined as their understanding of themselves in relation to their professional roles, values, and contexts (Beijaard et al., 2004)—has long been recognized as a critical determinant of pedagogical practice and classroom climate. Empirical evidence suggests that teachers’ professional identity profoundly shapes their teaching approaches, thereby influencing curriculum reform, classroom instruction, and student achievement (Caihong, 2011). Moreover, teachers with a well-developed professional identity tend to exhibit stronger commitment, deeper emotional engagement, and greater reflective capacity in their professional practice (Farrell, 2011). These findings imply that teachers’ professional identity may exert a direct and meaningful influence on students’ language grit. Supporting this perspective, Datu (2021) called for more research examining how interpersonal, contextual, and social factors relate to learners’ grit. Similarly, in their systematic review of 32 studies, Zhao and Wang (2023b) highlighted the need for further investigations into language grit across diverse contexts, participant groups, and research scopes, particularly to identify its underlying sources.

This notable gap is particularly pertinent to the Libyan educational context, where English language teaching continues to face systemic challenges. The education system in Libya remains largely exam-oriented, emphasizing grammatical accuracy and rote learning over communicative competence (Orafi and Borg, 2009). These conditions may limit students’ opportunities for meaningful interaction and intrinsic engagement with the language, thereby diminishing their motivation and perseverance. Consequently, the interaction between students’ language grit and teachers’ professional identity within this context remains an important but underexplored area of inquiry.

Accordingly, the present study aims to investigate whether a significant link exists between students’ language grit and their teachers’ professional identity in the Libyan context. More specifically, the study was conducted to explore the following research question: Is there a significant correlation between teachers’ professional identity and students’ language grit?. The findings are expected to contribute to a more nuanced understanding of the affective dimensions of language learning and teaching, particularly in contexts characterized by structural and pedagogical constraints.

## 2 Literature review

Research on teacher professional identity in the EFL context has become a focal point due to shifts in pedagogical roles driven by educational reforms and sociological factors in recent years. Agila (2023) conducted a study indicating that lecturing practices and institutional dynamics influence the reconstruction of EFL teachers’ professional identity, particularly the development of teacher self-concept in relation to curriculum demands and student engagement. Student grit—defined as perseverance and passion for long-term goals—enhances academic resilience and contributes significantly to success in language learning (Duckworth et al., 2007). Research into student grit within Libyan EFL settings remains in its infancy, despite the concept being extensively explored in Western educational contexts. This gap motivated the authors to investigate the topic, aiming to contribute insights into how grit manifests among Libyan EFL learners.

### 2.1 Theoretical framework

We frame our study via the lens of Social Cognitive Theory (SCT) by Bandura (1986), which we believe is both meaningful and rational, as it emphasizes learning within a social context and highlights the dynamic interplay among personal, behavioral, and environmental factors. Grounding our study in SCT enables us to examine how teachers’ professional self-concept, pedagogical stance, and behavioral modeling influence students’ perseverance and long-term goal development (Zhou and Hou, 2025).

Social Cognitive Theory (SCT) mainly focuses on observational learning, where teacher–student relationships are culturally situated and pedagogically influential (Open Oregon Educational Resources, 2025). Students adopt their teachers as role models when they observe them demonstrating professional resilience, adaptive strategies, and a strong commitment to student success (Zhou and Hou, 2025). In such milieus, where students are continuously exposed to teacher behaviors, they not only acquire linguistic competence but also internalize attitudes and dispositions—such as grit—as part of their personal development (Huang et al., 2025).

Self-efficacy, as one of the core components of SCT, reinforces both teacher identity and student grit. Teachers who exhibit high confidence in their instructional roles demonstrate a meaningful professional identity that fosters students’ belief in their own competencies. A teacher who demonstrates a high level of resilience and acts as a culturally responsive educator helps students develop grit by reinforcing the value of their teacher’s behavior (Huang et al., 2025).

All these insights, aligned with SCT, confirm that teacher identity is a socially constructed and performative force that can shape students’ personal development. We ground our study in Social Cognitive Theory (SCT) because we believe that, within Libya’s evolving educational reforms and efforts toward linguistic empowerment—especially in the EFL context—this theoretical framework offers both conceptual depth and practical relevance. The outcomes of this interplay are expected to contribute meaningfully to the development of Libya’s evolving language policy in EFL education.

### 2.2 Grit

Research in second language acquisition (SLA) has increasingly drawn upon the principles of positive psychology to illuminate the emotional and motivational dimensions of language learning (Dewaele et al., 2019; MacIntyre and Mercer, 2014). As one of the central pillars of positive psychology (Seligman, 2012), grit, encompassing persistence and passion for long-term goals, has been gaining considerable research and scholarly attention in recent years since its development by Duckworth et al., 2007. Grit, according to Duckworth et al. (2007), consists of two key factors: sustained interest and persistent effort. Sustained interest refers to sustaining a steady passion for working toward long-term goals, even when there are difficulties and barriers, and persistent effort refers to the ability of a person to sustain effort and energy in a steady and continuous way for a very long period of time (Duckworth et al., 2007; Duckworth and Gross, 2014). In language learning, L2 grit is the domain-specific adaptation that captures sustained effort and consistent interest in second language learning tasks over time (Teimouri et al., 2022). Teimouri et al. (2022) note that grit has a key contribution toward perseverance and motivation when there are difficulties, and this becomes more pronounced when considered in terms of second language acquisition. Considering that language acquisition is a long-term process, grit is essential for achieving proficiency, as gritty students persist in their goals despite facing difficulties and disappointments (Duckworth and Gross, 2014). Language grit interfaces naturally with motivational constructs in SLA such as integrative/instrumental motivation traditions and self-determination theory (Deci and Ryan, 2000) insofar as perseverance is both motivated by internalized goals and supported by contextual affordances (Alamer, 2021b).

Past research (Alamer, 2021a; Khajavy et al., 2021; Teimouri et al., 2022; Teimouri et al., 2024; Zhang and Zhang, 2023) has looked into language grit and its role in second and/or foreign language learning. However, these studies painted inconsistent pictures. For example, Teimouri et al. (2022) found that overall L1-Persian EFL learners’ L2 grit and perseverance of effort were significantly and positively correlated with all language achievement measures, while persistence of interest showed non-significant positive association with the achievement measures. Zhang and Zhang (2023) further explored the relationship between students’ grit and their cognitive abilities in English as a foreign language in predicting writing performance. They found that perseverance had a significant contribution to L2 writing in the argumentative task, while both perseverance of effort and persistence of interest had significant impacts on the narrative task. Teimouri et al. (2024) in their study found language grit to be highly related to language achievement. However, Alamer (2021a) conducted research on the relationship between Saudi undergraduate learners’ second-language English vocabulary knowledge and their grit and concluded that there was no relationship between second-language English vocabulary knowledge and grit. Similarly, Khajavy et al. (2021) reported no relation between university students’ L2 achievement and grit.

Although the above-mentioned studies have focused only on individual dispositions and considered language grit as a relatively stable trait, limited research (Bai and Zheng, 2024; Derakhshan et al., 2025; Hejazi and Sadoughi, 2022; Shen and Guo, 2022; Tang and Zhu, 2024; Yuan, 2022) has examined teacher support and respect, teacher confirmation and emotional support, teacher-student rapport and teacher stroke, teachers’ enthusiasm, support, appreciation, and their impact on learners’ language grit. These studies found a positive correlation among such variables. In their 2022 study investigating the impact of teacher support and respect on grit among learners majoring in English in China, Shen and Guo (2022) found that both teacher support and respect significantly predicted augmented grit levels in these students. The researchers suggest that positive teacher-student relationships can foster greater perseverance and passion for long-term learning goals in EFL contexts. Hejazi and Sadoughi (2022) further revealed in their study that L2 grit was found to be directly and positively predicted by teacher support and their findings underscore the vital role teachers play in creating a supportive learning environment, motivating students, and encouraging them to invest significant effort in their studies while fostering their interest, passion, persistence, and dedication. Yuan (2022) concluded that teacher stroke and teacher-student relationship were significantly related to grit among English learners in China. In the same vein, Tang and Zhu (2024) found a positive correlation between language grit and teacher support, implying that students receiving support from their teachers exhibit high level of grit. Derakhshan et al. (2025) discovered that teachers’ enthusiasm, support, and appreciation were found be related to language learners’ grit. Recently, Bai and Zheng (2024) uncovered that second language grit was found to be significantly correlated with both teacher confirmation and emotional support. Building on such findings, we argue that language grit would significantly associate with EFL teachers’ professional identity.

### 2.3 Teacher professional identity

Teachers, as central stakeholders in the educational system, play a pivotal role in shaping instructional effectiveness and student achievement (Yuan, 2022). Over the past two decades, increasing scholarly attention has been directed toward understanding teachers’ professional identity—a construct that reflects how teachers perceive themselves, negotiate their roles, and construct meaning within their professional and sociocultural contexts. This growth of interest stems from the recognition that teacher identity is dynamic and context-sensitive, constantly shaped by individual beliefs, institutional expectations, and broader social and cultural forces (Beauchamp and Thomas, 2009; Beijaard et al., 2000; Rushton et al., 2023). A coherent and positive sense of professional identity enables teachers to act with confidence and agency, engage in reflective practice, and adapt effectively to pedagogical and policy change. Furthermore, a well-developed professional identity tends to foster supportive teacher–student relationships and enrich classroom interactions, thereby enhancing learners’ motivation, engagement, and academic achievement.

Caihong (2011) notes that teachers’ identity influences their instructional decisions and classroom behaviors, which in turn affect curriculum reform, instructional quality, and learner outcomes. Similarly, Deniz (2022) and Yang (2023) contend that instructors’ sense of identity shapes their perceptions of appropriate pedagogical conduct and significantly impacts their contributions to educational advancement. Professional identity is a multifaceted and evolving construct, influenced by teachers’ values, beliefs, experiences, and sociocultural backgrounds. Within the field of language education, researchers have underscored the crucial role of teacher identity in shaping pedagogical knowledge and professional growth (Barahona and Ibaceta-Quijanes, 2020), highlighting the need for a comprehensive understanding that integrates both personal and contextual dimensions.

Empirical investigations have examined teacher professional identity from multiple perspectives. Pennington and Richards (2016) argue that teacher identity develops through an ongoing process of negotiation between personal beliefs, professional experiences, and institutional contexts—each reinforcing the other, as experiences shape beliefs and values influence pedagogical choices. Also, Sheybani and Miri (2019) found a strong relationship between English language instructors’ professional identity and their critical thinking skills, demonstrating that cognitive ability is a key component in shaping teachers’ professional perceptions and competence.

Similarly, Derakhshan et al. (2020) revealed that a well-developed professional identity enhances teachers’ sense of success, efficacy, and autonomy, affirming that effective teaching is deeply rooted in teachers’ self-perception and professional belief. Jia and Derakhshan (2023) further confirmed that professional identity is closely linked to job satisfaction and professional commitment among Chinese EFL teachers, showing that teachers with stronger professional identities display higher motivation, autonomy, and dedication to teaching. Recently, Alam and Asmawi (2023) examined identity salience among multi-subject generalist teachers and showed how identity emerged through negotiation, adaptation, and social endorsement — demonstrating that identity salience varies with professional experiences and institutional affordances. Such findings reinforce that teacher identity is fluid and responsive to systemic, institutional, and interpersonal forces. Overall, these findings emphasize that professional identity is integral to teachers’ emotional well-being, professional fulfillment, and instructional effectiveness.

Building on this evidence, we argue that teachers’ professional identity may extend its influence to students’ emotional and motivational attributes—particularly their language grit, defined as sustained perseverance and passion for long-term language learning goals. Teachers with a strong sense of professional identity are more likely to create positive, supportive, and motivating classroom environments that may promote student engagement and persistence. As Burroughs et al. (2019) state, one of the essential responsibilities of teachers is to cultivate constructive classroom climates that enhance learners’ academic outcomes and foster enduring motivation toward learning.

## 3 Methodology

### 3.1 Research design

This study is grounded in the positivist philosophical paradigm, which assumes that social phenomena such as teachers’ professional identity and students’ grit can be objectively observed, measured, and analysed to identify relationships among variables (Park et al., 2020). The positivist stance, based on realist ontology, emphasizes empirical observation, association, and statistical generalization. This orientation aligns with the present study’s goal of determining links between measurable psychological constructs rather than exploring subjective experiences. Accordingly, this research adopts a cross-sectional, quantitative, correlational (non-experimental) design, suitable for examining the strength and direction of relationships among naturally occurring variables without manipulation or random assignment (Creswell and Creswell, 2018). Both constructs were measured and Pearson’s correlation was employed to assess associations. Correlational designs are well established in educational and psychological research for exploring empirical correlations between social and psychological variables (Ary et al., 2019). Hence, the philosophical and methodological choices of the study — a positivist stance and a correlational quantitative design — are well aligned with the research aim of examining relationships between students’ L2 grit and teachers’ professional identity in the Libyan educational context.

### 3.2 Participants and sampling procedure

Participants were recruited through a convenience sampling method, whereby an online questionnaire was distributed to individuals who were accessible and willing to participate (Etikan et al., 2016). This approach was adopted because it allowed the researchers to efficiently reach English students and teachers within the practical constraints of time and resources.

The sample comprised 240 undergraduate English-major students (aged 18–24 years) and 42 EFL instructors (teaching experience ranging from 1 to 20 years). All participants were affiliated with two public universities located in Aljabel Alakhder, eastern Libya. Both universities offer the same undergraduate degree in English as a Foreign Language (EFL) and share comparable curricular structures, academic standards, and institutional missions. The student age range (18–24 years) reflects the typical undergraduate demographic within Libyan higher education. The teaching experience range (1–20 years) represents the spectrum of early-career to expert instructors within the regional university context. The observed imbalance between students (*n* = 240) and instructors (*n* = 42) reflects the hierarchical nature of university settings, where each instructor typically teaches multiple students.

### 3.3 Instruments

In the present study, two well-established instruments were employed: the Language Grit Scale (Teimouri et al., 2022) and the English Language Teacher Professional Identity Scale (Hashemi et al., 2021). Both instruments were originally developed and validated within English as a Second or Foreign Language (ESL/EFL) contexts, providing robust evidence of content validity and construct reliability in their initial studies. Given that these measures were theoretically grounded in second language learning and teaching and empirically supported across EFL/ESL populations, no substantial adaptations were necessary for their use in the Libyan context. Nonetheless, a preliminary review was undertaken to ensure that all items were clear, culturally appropriate, and easily understood by Libyan EFL participants. Reliability analyses conducted with the current sample further confirmed the internal consistency of both instruments, as reported in Table 1. Details of the abovementioned instruments are presented in the subsequent sections.

**TABLE 1:** Results of Cronbach alpha.

Scale	Subscales	Number of items	Cronbach alpha
Language grit	Perseverance of effort	5	0.86
	Consistence of interest	4	0.42
	Total	9	0.71
Teacher professional identity	Creating a relaxed learning atmosphere	4	0.97
	Tendency to impart knowledge and experience	5	0.96
	Having respectful behavior	3	0.95
	Having the ability to develop/select EFL materials	4	0.93
	Having management skills	4	0.89
	Having error correction skills	3	0.90
	Having communication skills	3	0.95
	Creating an effective teaching environment	3	0.91
	Having the tendency to develop professionally	2	0.85
	Familiarity with target language and culture	2	0.91
	Serving as an effective role model	4	0.89
	Valuing L1 culture	2	0.80
	Concerned about students’ ability and development	3	0.82
	Total	42	0.98

#### 3.3.1 L2 grit

The questionnaire on language grit developed by Teimouri et al. (2022) was used to assess the language grit of the students. This scale follows a five-point Likert-type format, with responses ranging from 1 strongly disagree to 5 strongly agree. The questionnaire consists of 9 items, divided into two categories: perseverance of effort (5 items) and consistency of interest (4 items).

#### 3.3.2 English language teacher professional identity

The English Language Teacher Professional Identity Scale, developed by Hashemi et al. (2021), contains 42 items and utilizes a 5-point Likert scale, with responses ranging from 1 strongly disagree to 5 strongly agree was employed to assess the professional identity of English language teachers. This scale includes 13 sub-scales: creating a relaxed learning atmosphere (4 items), imparting knowledge and experience (5 items), having respectful behavior (3 items), developing/selecting EFL materials (4 items), having management skills (4 items), having error correction skills (3 items), having communication skills (3 items), creating teaching environment (3 items), the tendency to professional development (2 items), familiarity with target language and culture (2 items), serving as an effective role model (4 items), valuing L1 culture (2 items), and being concerned about students’ ability (3 items).

### 3.4 Dada collection and analysis

Ethical approval for this study was obtained from the research ethics committees of the two participating government universities. Following institutional permission, two separate online links were created for the two questionnaires: The link for Language Grit Scale targeting students and the one for English Language Teacher Professional Identity Scale targeting instructors were posted on the universities’ official Facebook pages throughout the academic year (2023–2024; 4 Nov.–24 Nov.). The initial page of the two online questionnaires contained a consent form that participants were required to agree prior to responding to the questions and they were informed of the survey’s purpose, that there were no right or wrong answers, and that their participation was entirely voluntary. The online links remained active for 3 weeks to allow participants to complete the questionnaires at their convenience and only 240 students and 42 instructors were responded to the web-based surveys from 4 November to 24 November.

Although online recruitment may limit representativeness by primarily engaging individuals with internet access or social media presence, it was considered appropriate for the target population in this study since the participants are English major university instructors and students, who have generally access to social media as well as are active online. Online recruitment has been increasingly recognized as an efficient and valid method for data collection in educational research (Bakla et al., 2013). To enhance diversity and minimize potential sampling bias, participants were recruited through official Facebook pages of the two universities.

The datasets for both students and instructors were examined for incomplete or missing responses prior to analysis. The overall rate of missingness was low and did not affect the integrity of the findings. Missing data were controlled using pairwise deletion, which allows for the inclusion of all available data points in each individual analysis without discarding entire cases due to partial non-response. This approach was chosen to preserve statistical power while minimizing bias, given the low rate of missingness observed in our dataset (less than 5%). Data were then analyzed using IBM SPSS Statistics version 27.0. This version was selected since it offers a comprehensive set of tools apt for descriptive, inferential, and multivariate analyses, and it was the licensed version available at the time the study was conducted. Descriptive statistics, including means and standard deviations, were employed to summarize teachers’ perceptions of their professional identity in English language teaching and students’ perceptions of their language learning grit. Pearson’s correlation analysis was subsequently conducted to examine the relationship between English language teachers’ professional identity and their students’ grit levels.

## 4 Results

Table 1 presents the data derived from the Cronbach’s Alpha analyses. As shown, the reliability analyses for both scales demonstrated internal consistency as a whole, with a Cronbach’s alpha coefficient of (α = 0.98) for the Teacher Professional Identity Scale and (α = 0.71) for the Language Grit Scale. These numbers suggest that the internal consistency of the two scales is good as it is greater than the recommended value of above 0.70 (Tavakol and Dennick, 2011). All the subscales of the two questionnaires gained internal consistency, except one of the sub-scales of language grit, consistent of interest, yielded lower reliability (α = 0.42). This is consistent with previous findings and reflects the inherent variability of this trait (Derakhshan et al., 2025).

Table 2 presents the descriptive statistics of sub-scales of teacher professional identity and language grit. Among 13 sub-scales of teacher professional identity, *Having management skills* (4.04) has the highest mean score, while *Familiarity with target language and culture* (3.03) has the lowest mean score. Of sub-scales of language grit, *Perseverance of effort* (3.85) has the highest mean score, whereas *Consistency of interest* (2.77) has the lowest mean score.

**TABLE 2:** Descriptive statistics of teachers’ professional identity and students’ language grit.

Teachers’ professional identity	N	Min	Max	Mean	SD
Creating a relaxed learning atmosphere	42	1	5	3.95	1.06
Tendency to impart knowledge and experience	42	1	5	3.96	0.87
Having respectful behavior	42	1	5	3.93	0.92
Having the ability to develop/select EFL materials	42	1	5	3.94	0.83
Having management skills	42	1	5	4.04	0.75
Having error correction skills	42	1	5	4.03	0.92
Having communication skills	42	1	5	3.92	0.98
Creating an effective teaching environment	42	1	5	3.97	0.89
Having the tendency to develop professionally	42	1	5	4.01	1.11
Familiarity with target language and culture	42	1	5	3.03	1.46
Serving as an effective role model	42	1	5	3.99	0.89
Valuing L1 culture	42	1	5	4.00	0.89
Concerned about students’ ability and development	42	1	5	3.99	0.75
**Students’ language grit**					
Perseverance of effort	240	1	5	3.85	0.91
Consistency of interest	240	1	5	2.77	0.74

Tables 3, 4 present the Pearson correlation analysis examining the relationship between students’ language grit and English language teachers’ professional identity. In Table 3, the findings indicate no significant positive correlation (*r* = 0.236) between teachers’ overall professional identity and students’ overall language grit. In Table 4, no significant association was identified between specific components of language grit and teachers’ professional identity.

**TABLE 3:** Overall Correlation between professional identity and students’ language grit.

Variable	Professional identity	Language grit
Professional identity	1	0.236
Language grit	0.236	1
Sig. (2-tailed)	0.133	–
N	42	240

**TABLE 4:** Correlation between components of English language teachers’ identity and students’ language grit.

N	Sub-constructs	1	2	3	4	5	6	7	8	9	10	11	12	13	14	15
1	Creating a relaxed learning atmosphere	1.00														
2	Imparting knowledge and experience	0.87**	1.00													
3	Having respectful behavior	0.82**	0.86**	1.00												
4	Developing/selecting EFL materials	0.68**	0.75**	0.75**	1.00											
5	Having management skills	0.82**	0.82**	0.83**	0.79**	1.00										
6	Having error correction skills	0.75**	0.76**	0.80**	0.79**	0.87**	1.00									
7	Having communication skills	0.81**	0.83**	0.85**	0.86**	0.78**	0.81**	1.00								
8	Creating teaching environment	0.74**	0.76**	0.73**	0.75**	0.76**	0.79**	0.85**	1.00							
9	The tendency to professional development	0.76**	0.76**	0.69**	0.71**	0.72**	0.73**	0.75**	0.90**	1.00						
10	Familiarity with target language and culture	0.13	0.10	0.23	0.08	0.03	0.29	0.19	0.23	0.15	1.00					
11	Serving as an effective role model	0.72**	0.75**	0.84**	0.77**	0.80**	0.77**	0.78**	0.72**	0.67**	0.10	1.00				
12	Valuing L1 culture	0.76**	0.74**	0.76**	0.79**	0.77**	0.78**	0.85**	0.80**	0.70**	0.23	0.81**	1.00			
13	Being concerned about students’ ability	0.58**	0.62**	0.61**	0.71**	0.61**	0.67**	0.62**	0.67**	0.63**	0.31*	0.60**	0.79**	1.00		
14	Perseverance of effort	0.25	0.24	0.18	0.09	0.27	0.16	0.21	0.26	0.30	−0.01	0.15	0.03	−0.17	1.00	
15	consistency of interest	0.06	0.20	0.12	0.30	0.18	0.23	0.18	0.24	0.25	0.13	0.22	0.15	0.19	0.18**	1.00

**Correlation is significant at the 0.01 level (2-tailed). *Correlation is significant at the 0.05 level (2-tailed).

## 5 Discussion

The current study aimed at investigating the relationship between the professional identity of English language instructors and students’ language grit. To achieve this goal, the following research question was addressed: Is there a significant relationship between English language teachers’ professional identity and their students’ language grit levels? A quantitative, correlational (non-experimental) design was used to explore this question. The results revealed no statistically significant correlation (*r* = 0.236) between overall professional identity of English language teachers and overall student language grit, and between the sub-constructs of language grit and the sub-scales of English language teachers’ professional identity.

Interpreting these results through the lens of Social Cognitive Theory (SCT) may provide a deeper understanding of the observed patterns. SCT posits that learning is a socially mediated process resulting from the mutual interaction of personal, behavioral, and environmental factors (Bandura, 1986). Within this framework, teachers act as powerful models whose professional behaviors and attitudes shape students’ beliefs, motivation, and perseverance through observational learning (Zhou and Hou, 2025; Huang et al., 2025). The lack of association found in this study suggests that while teacher modeling is more likely to wield some influence, its effect may be weakened by contextual and motivational factors specific to the Libyan EFL environment.

SCT also highlights the centrality of self-efficacy in learning. Teachers with a strong professional identity typically display high confidence in their instructional roles, which can enhance students’ belief in their own capabilities. Yet, the absence of a significant relationship in this study suggests that students’ self-efficacy—and consequently their grit—may be shaped more strongly by external conditions such as socioeconomic status, family expectations, and institutional constraints than by teacher identity. This aligns with SCT’s assertion that behavioral outcomes are context-dependent and mediated by both environmental affordances and personal belief systems.

Although teachers’ professional identity is often considered crucial to students’ academic success in higher education (Baxter, 2012), and a positive correlation was found between language grit and teacher support, teacher respect, emotional support, teacher confirmation, teacher-student rapport and teacher stroke, teachers’ enthusiasm and appreciation (Bai and Zheng, 2024; Derakhshan et al., 2025; Hejazi and Sadoughi, 2022; Shen and Guo, 2022, Tang and Zhu, 2024; Yuan, 2022), our findings do not provide strong evidence of a significant relationship between students’ language grit and professional identity of teachers. A potential explanation for the discrepancy between our findings and those in the existing literature is that language grit—which embodies perseverance and passion for long-term goals—is predominantly driven by internal motivational factors rather than external influences such as the professional identity of the teacher. This finding confirms what Li (2024) states that grit is a type of internal motivational resource, which, in turn, may not be influenced by external factors such as teachers’ professional identity. Moreover, our findings align with Solhi et al. (2025), who identified three distinct profiles—goal-oriented optimists, self-regulated strategists, and passionate devotees—underscoring the multifaceted and dynamic nature of grit shaped by learners’ motivation, self-regulation, and personal drive.

The non-significant link found between English language teachers’ professional identity and students’ language grit in the present study can be attributed to a variety of reasons. Firstly, students’ language grit may largely be shaped by other external factors, such as personal mindsets. Previous research has found that students’ growth mindsets are significantly related to their language grit (Kırmızı et al., 2023; Zarrinabadi et al., 2022). Students with a growth mindset—those who consider challenges as opportunities to improve—are more likely to demonstrate perseverance, regardless of teacher-related factors. Secondly, in the Libyan context, external motivators—such as career opportunities and societal expectations—often have a crucial role in language learning than teacher-related factors. Students’ language grit may not correlate with their teachers’ professional identity when they regard English as a necessity for educational success rather than a subject of personal interest. In many EFL settings, extrinsic factors, including employment prospects and educational achievements, are more influential in shaping students’ perseverance than direct teacher influence (Ushioda, 2013). Lastly, in Libya, English proficiency is primarily perceived as an urgent need for academic and career progression rather than a quest driven by intrinsic passion. This perspective may explain why students remain committed to language learning without necessarily linking their language grit to their English language teachers’ professional identity.

Furthermore, while teachers’ professional identity undeniably plays a pivotal role in the educational experience, its impact may not always be directly reflected in student outcomes such as language grit—particularly in contexts where students are driven more by instrumental rather than integrative motivation. As Deci and Ryan (2000) emphasize in their Self-Determination Theory, intrinsic motivation—which is essential for fostering grit—is strongly associated with three fundamental psychological needs: competence, autonomy, and relatedness. In settings like Libya, where language learning is primarily viewed through a lens of practicality rather than passion, these intrinsic needs may be less influenced by the teacher’s professional identity. Instead, students may be more motivated by external rewards such as grades, job opportunities, and social status.

In light of these findings, it becomes clear that while teachers’ professional identity may influence certain aspects of student engagement, context matters significantly when considering its role in promoting student outcomes such as language grit. External factors—including societal pressures, economic considerations, and personal mindset—can often play a more decisive role in shaping student perseverance and grit than the teacher’s identity alone. Therefore, it is crucial to consider the broader sociocultural and educational environment when examining the relationship between teachers and students’ motivational attributes.

### 5.1 Implications

#### 5.1.1 Theoretical implications

These findings are consistent with Social Cognitive Theory (SCT) while offering an opportunity to refine and extend its framework. They suggest that the mechanisms through which teacher identity shapes student grit are likely indirect and multifaceted, influenced by a combination of instructional practices, environmental conditions, and socio-cultural dynamics. The current study also indicates that these relationships may depend on contextual factors such as teacher autonomy, institutional support, and classroom climate. This nuance advances existing theory by implying that teacher identity alone may not be sufficient to predict learners’ perseverance, particularly in settings where pedagogical interaction is limited. Consequently, future research—especially within the Libyan context—should investigate these complex dynamics more deeply. Qualitative or mixed-methods studies could help uncover the subtle interconnections between teacher identity, contextual factors, and student grit, ultimately identifying the key aspects of teacher behavior and environmental conditions that most effectively nurture perseverance in learners.

#### 5.1.2 Pedagogical implications

The findings of this study suggest some important pedagogical implications. To begin with, teachers must prioritize autonomous learning strategy development by imparting self-regulation, goal-setting, and perseverance-based activities to enhance students’ intrinsic motivation. As teacher professional identity is not related to student language grit, programs in relation to teacher development should shift toward arming teachers with strategies enhancing grit in students, such as motivation activities and metacognitive training. Curriculum planners must also integrate project-based and inquiry-based learning activities that foster long-term perseverance, as student grit seems to be more shaped by wider instruction systems than by the professional identity of teachers. Moreover, as peripheral factors, including institutional challenges and socioeconomic conditions, could hold a more important role in defining student grit, further research should examine these to inform targeted interventions. Programs related to teacher training must also integrate grit-based pedagogy, focusing on resilience training, a growth mindset development, and reframing failure as an opportunity to learn, not as a failure. Finally, systemic reforms must be implemented by policymakers, including learning environment improvement, the deemphasis on exam-oriented education, and establishment of meaningful, long-term language learning projects that enhance the language grit of students. By taking a context-specific and all-inclusive perspective, such pedagogical recommendations can better facilitate grit development in EFL students in Libya and other EFL contexts.

### 5.2 Limitations and further research

Although this study holds valuable insights into the correlation between students’ language grit and English language teachers’ professional identity, suggesting that such identity is an insignificant predictor of students’ language grit, there are some limitations that should be acknowledged. Firstly, the study adopted a cross-sectional, quantitative, correlational (non-experimental) research design, collecting data at a single point in time and does not explicate potential changes in students’ language grit or teachers’ professional identity over time. Future research using a longitudinal approach would provide a more comprehensive understanding of how these variables interact and evolve throughout the learning process. Secondly, the study was conducted within the Libyan educational context, limiting the generalizability of the results to other EFL settings. Institutional, cultural, and socio-economic differences across different regions could have an impact on the correlation between students’ language grit and teachers’ professional identity in unique ways. Finally, while this study explored the association between teachers’ professional identity and students’ language grit, it did not investigate possible mediating or moderating factors, such as classroom environment, teaching methodologies, or students’ learning strategies. Future research should incorporate such variables to achieve deeper insights into the factors that affect students’ perseverance in language learning.

## Data Availability

The original contributions presented in this study are included in this article/supplementary material, further inquiries can be directed to the corresponding author.

## References

[B1] Agila N. S. (2023). The impact of lecturing on reconstructing teachers’ professional identity: A case study on four libyan efl in-service teachers teaching at the college of languages and translation, Zaytuna University. *Bani Walid J. Humanities Appl. Sci.* 8 436–459.

[B2] Alam M. S. Asmawi A. (2023). Understanding identity salience and English as a Foreign Language teaching practices: A case study of multi-subject generalist teachers. *Rev. Educ.* 11:e3424. 10.1002/rev3.3424

[B3] Alamer A. (2021a). Grit and language learning: Construct validation of L2-Grit scale and its relation to later vocabulary knowledge. *Educ. Psychol.* 41 544–562. 10.1080/01443410.2020.1867076

[B4] Alamer A. (2021b). Grit and motivation from a self-determination theory perspective in second language learning. *Front. Psychol.* 12:721708. 10.3389/fpsyg.2021.721708

[B5] Ary D. Jacobs L. C. Irvine C. K. S. Walker D. (2019). *Introduction to research in education*, 10th Edn. Boston, MA: Cengage Learning.

[B6] Bai S. Zheng W. (2024). Associations between teacher confirmation, emotional support, and Chinese EFL learners’ grit: Sequential mixed methods. *Perceptual Motor Skills* 131 1958–1983. 10.1177/00315125241272634 39126658

[B7] Bakla A. Çekiç A. Köksal O. (2013). Web-based surveys in educational research. *Int. J. Acad. Res. Part B* 5 5–13. 10.7813/2075-4124.2013/5-1/B.1

[B8] Bandura A. (1986). *Social foundations of thought and action: A social cognitive theory.* Englewood Cliffs, NJ: Prentice-Hall.

[B9] Barahona M. Ibaceta-Quijanes X. (2020). Neither fish nor fowl: The contested identity of teachers of English in an EFL context. *RELC J.* 51 347–363. 10.1177/0033688219847315

[B10] Baxter J. A. (2012). The impact of professional learning on the teaching identities of higher education lecturers. *Eur. J. Open Distance E-Learning* 2012 1–11.

[B11] Beauchamp C. Thomas L. (2009). Understanding teacher identity: An overview of issues in the literature and implications for teacher education. *Cambridge J. Educ.* 39 175–189. 10.1080/03057640902902252

[B12] Beijaard D. Meijer P. C. Verloop N. (2004). Reconsidering research on teachers’ professional identity. *Teach. Teach. Educ.* 20 107–128. 10.1016/j.tate.2003.07.001

[B13] Beijaard D. Verloop N. Vermunt J. D. (2000). Teachers’ perceptions of professional identity: An exploratory study from a personal knowledge perspective. *Teach. Teach. Educ.* 16 749–764. 10.1016/S0742-051X(00)00023-8

[B14] Burroughs N. Gardner J. Lee Y. Guo S. Touitou I. Jansen K. (2019). “A review of the literature on teacher effectiveness and student outcomes,” Burroughs N. Gardner J. Lee Y. Guo S. Touitou I. Jansen K. (Eds.) in *Teaching for excellence and equity: Analyzing teacher characteristics, behaviors and student outcomes with TIMSS*, (Cham: Springer), 7–17. 10.1007/978-3-030-16151-4_2

[B15] Caihong H. A. (2011). Changes and characteristics of EFL teachers’ professional identity: The cases of nine university teachers. *Chinese J. Appl. Linguistics* 34 3–21. 10.1515/cjal.2011.001

[B16] Creswell J. W. Creswell J. D. (2018). *Research design: Qualitative, quantitative, and mixed methods approaches*, 5th Edn. Thousand Oaks, CA: Sage Publications.

[B17] Datu J. A. D. (2021). Beyond passion and perseverance: Review and future research initiatives on the science of grit. *Front. Psychol.* 11:545526. 10.3389/fpsyg.2020.545526 33584397 PMC7873055

[B18] Deci E. L. Ryan R. M. (2000). The “What” and “Why” of goal pursuits: Human needs and the self-determination of behavior. *Psychol. Inquiry* 11 227–268. 10.1207/S15327965PLI1104_01

[B19] Deniz Ü (2022). What should we understand from teachers’ professional identity? An overview of the literature. *J. Innov. Res. Teach. Educ.* 3 113–124. 10.29329/jirte.2022.464.4

[B20] Derakhshan A. Coombe C. Arabmofrad A. Taghizadeh M. (2020). Investigating the effects of English language teachers’ professional identity and autonomy in their success. *Issues Lang. Teach.* 9 1–28. 10.22054/ilt.2020.52263.496

[B21] Derakhshan A. Solhi M. Azari Noughabi M. (2025). An investigation into the association between student-perceived affective teacher variables and students’ L2-grit. *J. Multilingual Multicult. Dev.* 46 798–814. 10.1080/01434632.2023.2212644

[B22] Dewaele J.-M. Chen X. Padilla A. M. Lake J. (2019). The flowering of positive psychology in foreign language teaching and acquisition research. *Front. Psychol.* 10:2128. 10.3389/fpsyg.2019.02128 31607981 PMC6769100

[B23] Duckworth A. L. Gross J. J. (2014). Self-control and grit: Related but separable determinants of success. *Curr. Dir. Psychol. Sci.* 23 319–325. 10.1177/0963721414541462 26855479 PMC4737958

[B24] Duckworth A. L. Peterson C. Matthews M. D. Kelly D. R. (2007). Grit: Perseverance and passion for long-term goals. *J. Pers. Soc. Psychol.* 92 1087–1101. 10.1037/0022-3514.92.6.1087 17547490

[B25] Etikan I. Musa S. A. Alkassim R. S. (2016). Comparison of convenience sampling and purposive sampling. *Am. J. Theoretical Appl. Stat.* 5 1–4. 10.11648/j.ajtas.20160501.11

[B26] Farrell T. S. C. (2011). Exploring the professional role identities of experienced ESL teachers through reflective practice. *System* 39 54–62. 10.1016/j.system.2011.01.012

[B27] Hashemi M. R. Karimi M. N. Mofidi M. (2021). Developing and validating an EFL teacher professional identity inventory: A mixed methods study. *Mextesol J.* 45 12–24. 10.61871/mj.v45n1-3

[B28] Hejazi S. Y. Sadoughi M. (2022). How does teacher support contribute to learners’ grit? The role of learning enjoyment. *Innov. Lang. Learn. Teach.* 17 593–606. 10.1080/17501229.2022.2098961

[B29] Huang T. Halim H. A. Jalaluddin I. B. Liu S. (2025). Beyond communicative competence: Investigating the impact of grit and classroom enjoyment on willingness to communicate. *Soc. Sci. Humanities Open* 12:101767. 10.1016/j.ssaho.2025.101767

[B30] Jia H. Derakhshan A. (2023). Chinese English teachers’ professional identity and commitment and their associations with their professional success. *Porta Linguarum* 9 47–64. 10.30827/portalin.vi2023c.29625

[B31] Khajavy G. H. MacIntyre P. D. Hariri J. (2021). A closer look at grit and language mindset as predictors of foreign language achievement. *Stud. Sec. Lang. Acquisition* 43 379–402. 10.1017/S0272263120000480

[B32] Kırmızı Ö Irgatoğlu A. Atalmış E. H. (2023). Examining the interplay between growth and fixed mindsets, L2 grit, and L2 motivational self-system of L2 learners. *Sage Open* 13 1–13. 10.1177/21582440231208997

[B33] Li M. (2024). The interplay between perceived teacher support, grit, and academic engagement among Chinese EFL learners. *Learn. Motivat.* 85:101965. 10.1016/j.lmot.2024.101965

[B34] MacIntyre P. D. Khajavy G. H. (2021). Grit in second language learning and teaching: Introduction to the special issue. *J. Psychol. Lang. Learn.* 3 1–6. 10.52598/jpll/3/2/1

[B35] MacIntyre P. D. Mercer S. (2014). Introducing positive psychology to SLA. *Stud. Sec. Lang. Learn. Teach.* 4 153–172. 10.14746/ssllt.2014.4.2.2

[B36] Open Oregon Educational Resources. (2025). *Social Cognitive Theory. In Educational Learning Theories*, 3rd Edn. Available online at: https://openoregon.pressbooks.pub/educationallearningtheories3rd/chapter/chapter-3-social-cognitive-theory-2/ (accessed October 25, 2025).

[B37] Orafi S. M. S. Borg S. (2009). Intentions and realities in implementing communicative curriculum reform. *System* 37 243–253. 10.1016/j.system.2008.11.004

[B38] Park Y. S. Konge L. Artino A. R. (2020). The positivism paradigm of research. *Acad. Med.* 95 690–694. 10.1097/ACM.0000000000003093 31789841

[B39] Pennington M. C. Richards J. C. (2016). Teacher identity in language teaching: Integrating personal, contextual, and professional factors. *RELC J.* 47 5–22. 10.1177/0033688216631219

[B40] Rushton E. Rawlings Smith E. Steadman S. Towers E. (2023). Understanding teacher identity in teachers’ professional lives: A systematic review of the literature. *Rev. Educ.* 11 e3417. 10.1002/rev3.3417

[B41] Seligman M. E. (2012). *Flourish: A visionary new understanding of happiness and well-being.* New York, NY: Simon and Schuster.

[B42] Shen Y. Guo H. (2022). Increasing Chinese EFL learners’ grit: The role of teacher respect and support. *Front. Psychol.* 13:880220. 10.3389/fpsyg.2022.880220 35592152 PMC9113390

[B43] Sheybani M. Miri F. (2019). The relationship between EFL teachers’ professional identity and their critical thinking: A structural equation modeling approach. *Cogent Psychol.* 6:1592796. 10.1080/23311908.2019.1592796

[B44] Solhi M. Thumvichit A. Teimouri Y. (2025). Examining the characteristics of gritty L2 learners: A Q methodology study. *Innov. Lang. Learn. Teach*. 1–22. 10.1080/17501229.2025.2573686

[B45] Tang L. Zhu X. (2024). Academic self-efficacy, grit, and teacher support as predictors of psychological well-being of Chinese EFL students. *Front. Psychol.* 14:1332909. 10.3389/fpsyg.2023.1332909 38259578 PMC10800794

[B46] Tavakol M. Dennick R. (2011). Making sense of Cronbach’s alpha. *Int. J. Med. Educ.* 2 53–55. 10.5116/ijme.4dfb.8dfd 28029643 PMC4205511

[B47] Teimouri Y. Plonsky L. Tabandeh F. (2022). L2 grit: Passion and perseverance for second-language learning. *Lang. Teach. Res.* 26 893–918. 10.1177/1362168820921895

[B48] Teimouri Y. Tahmouresi S. Tabandeh F. (2024). The interplay of mindsets, aptitude, grit, and language achievement: What role does gender play? *Stud. Sec. Lang. Acquisition* 46 1–24. 10.1017/S0272263124000330

[B49] Ushioda E. (2013). “Motivation and ELT: Looking ahead to the future,” in *International perspectives on motivation and language learning professional challenges*, ed. Ushioda E. (London: Palgrave Macmillan), 233–239.

[B50] Yang Y. (2023). Challenges in teachers’ professional identity development under the National teacher training programme: An exploratory study of seven major cities in mainland China. *Music Educ. Res.* 25 468–484. 10.1080/14613808.2023.2246136

[B51] Yuan L. (2022). Enhancing Chinese EFL students’ Grit: The impact of teacher stroke and teacher-student rapport. *Front. Psychol.* 12:823280. 10.3389/fpsyg.2021.823280 35126266 PMC8813776

[B52] Zarrinabadi N. Rezazadeh M. Mohammadabadi M. A. (2022). L2 grit and language mindsets as predictors of EFL learners’ attitudes toward effectiveness and value of CALL. *Comput. Assisted Lang. Learn.* 37 1726–1743. 10.1080/09588221.2022.2108061

[B53] Zhang C. Mao L. Li N. Gu X. (2022). Chinese EFL students’ social-emotional competence, grit, and academic engagement. *Front. Psychol.* 13:914759. 10.3389/fpsyg.2022.914759 35756262 PMC9231457

[B54] Zhang J. Zhang L. J. (2023). Examining the relationship between English as a foreign language learners’ cognitive ability and L2 grit in predicting their writing performance. *Learn. Instruction* 88:101808. 10.1016/j.learninstruc.2023.101808

[B55] Zhao X. Wang D. (2023a). Grit, emotions, and their effects on ethnic minority students’ English language learning achievements: A structural equation modelling analysis. *System* 113 1–37. 10.1016/j.system.2023.102979

[B56] Zhao X. Wang D. (2023b). Grit in second language acquisition: A systematic review from 2017 to 2022. *Front. Psychol.* 14:1238788. 10.3389/fpsyg.2023.1238788 37727745 PMC10506257

[B57] Zhou M. Hou Y. (2025). Teacher self-efficacy and grit as predictors of student perseverance and academic resilience. *BMC Psychol.* 13:468. 10.1186/s40359-025-02761-6 40325446 PMC12051330

